# Supplementary material for: Genetic separation of Brca1 functions reveal mutation-dependent Polθ vulnerabilities
Source: Nat Commun. 2023 Nov 24;14:7714. doi: 10.1038/s41467-023-43446-1 (PMC10673838; doi:10.1038/s41467-023-43446-1)
Supplement: Supplementary file 1 — Supplementary Information [file 41467_2023_43446_MOESM1_ESM.pdf]

Supplemental Figures

Supplementary Figure 1.

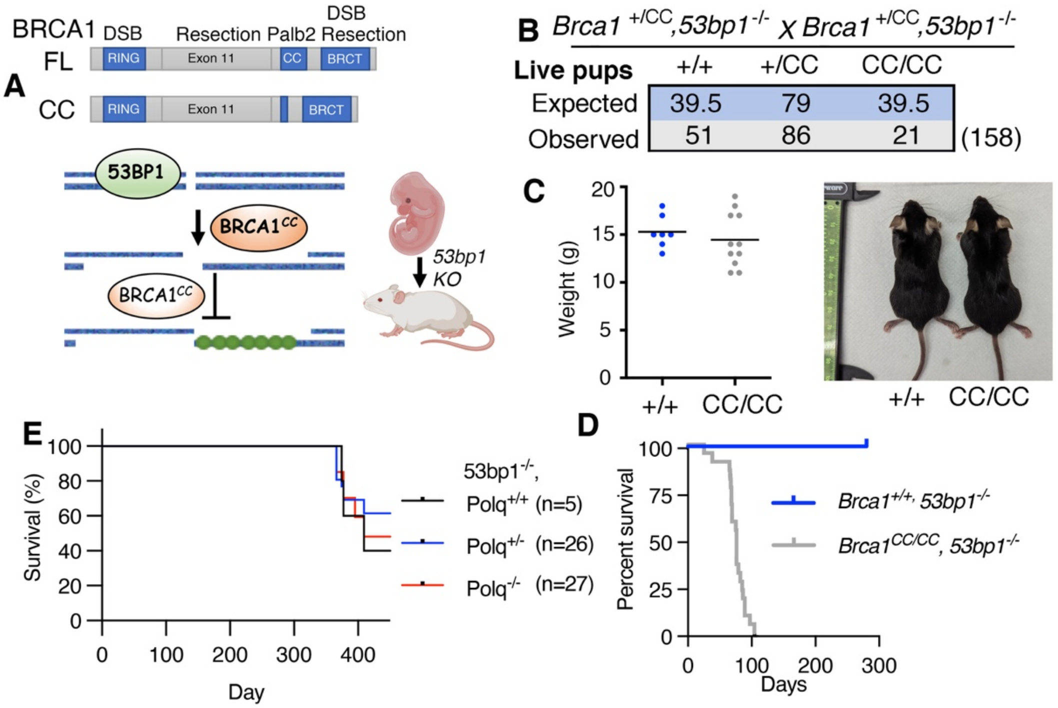

Supplementary Figure 1. *Brca1, 53bp1* and *Polq* mouse intercrosses

(**A**) Cartoon showing Brca1 full-length (FL) and the Brca1-CC proteins. In contrast to other *Brca1* alleles, the Brca1-CC protein is functional for resection, but defective for Rad51 loading. Therefore, the effect of *53bp1*$^{-/-}$, which hyperactivates resection, is less predictable for *Brca1*^{CC} mouse development. Created with BioRender.com

(**B**) *Brca1*$^{+/CC}$*, 53bp1*$^{-/-}$ are normal and are fertile, producing offspring with the genotypes as shown in the table compared to the expected Mendelian ratio. *Brca1*$^{CC/CC}$*, 53bp1*$^{-/-}$ mice are born at a sub-Mendelian ratio suggesting an incomplete rescue by *53bp1* KO.

(**C**) *Brca1*$^{CC/CC}$*, 53bp1*$^{-/-}$ and *Brca1*$^{+/+}$*, 53bp1*$^{-/-}$ littermates were weighed at 6 weeks old and were not significantly different in weight or appearance (p=0.4976 by unpaired, two-tailed *t*-test). Photograph of representative mice.

(**D**) Kaplan-Meier survival analyses of *Brca1*$^{+/+}$*, 53bp1*$^{-/-}$ and *Brca1*$^{CC/CC}$*, 53bp1*$^{-/-}$ littermates.

(**E**) Survival of*53bp1*$^{-/-}$ with *Polq*$^{+/+}$*, Polq*$^{+/-}$*, and Polq*$^{-/-}$ littermates were measured in a *53bp1*$^{-/-}$ background with no differences observed between genotypes. Source data are provided as a Source Data file.

Supplementary Figure 2.

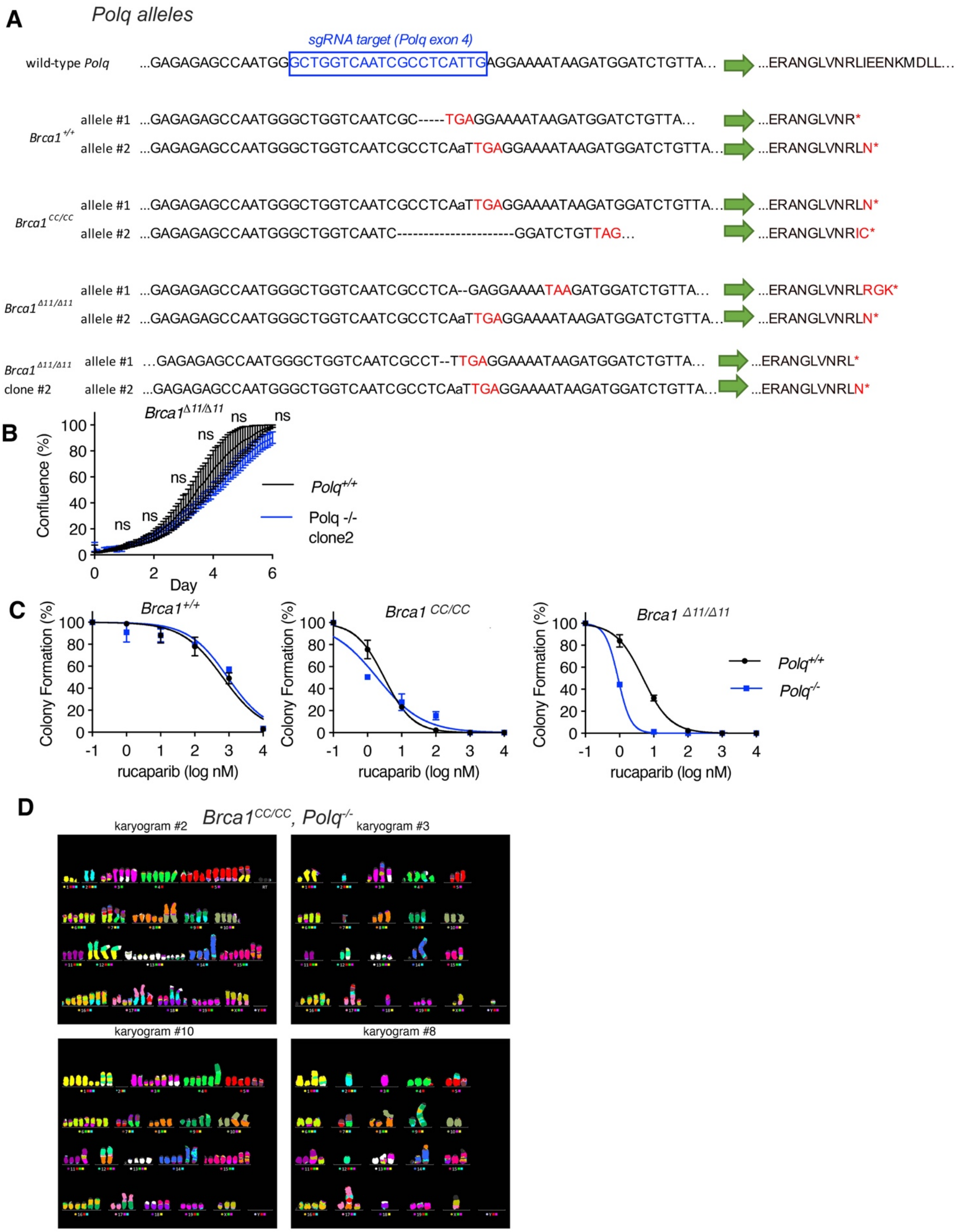

Supplementary Figure 2. Characterization of immortalized *Polq* KO mouse cell lines

(**A**) DNA was extracted from *Polq* KO clones from Fig.1d and mutation status confirmed by next-generation amplicon sequencing. The wild-type allele, sgRNA target and wild-type amino acid sequence are shown. Two variants were detected for each genotype at similar ratios. Each mutation results in a frameshift that causes a disruption to the amino acid sequence and early stop codon.

(**B**) Growth curves were obtained for $Polq^{-/-}$ clones and $Brca1^{\Delta11/\Delta11}$ MEFs clone#2 based on confluence measurements obtained using a Sartorius Incucyte imager. Confluence and S.E.M. of 3 independent experiments are shown. ns, not significant (p>0.05, unpaired, two-tailed t-tests).

(**C**) *Brca1*$^{+/+}$, *Brca1*$^{CC/CC}$ *and Brca1*$^{\Delta11/\Delta11}$ MEFs with control sgRNA targeting GFP or the *Polq* alleles described in A were seeded into increased concentrations of the PARPi rucaparib. Colony formation was assessed after 2 weeks.

(**D**) Karyograms after mFISH staining for independent *Brca1*$^{CC/CC}$*, Polq*$^{-/-}$ metaphases are shown. Despite originating from a single cell clone the varied abnormalities highlight the subclonal heterogeneity observed within *Brca1*$^{CC/CC}$*, Polq*$^{-/-}$ cells, potentially indicative of a high level of genome instability. Metaphase numbers correspond to the analysis shown in **Supplementary Data 3**. Karyogram #5 is shown in **Fig. 2d**. Source data are provided as a Source Data file.

Supplementary Figure 3.

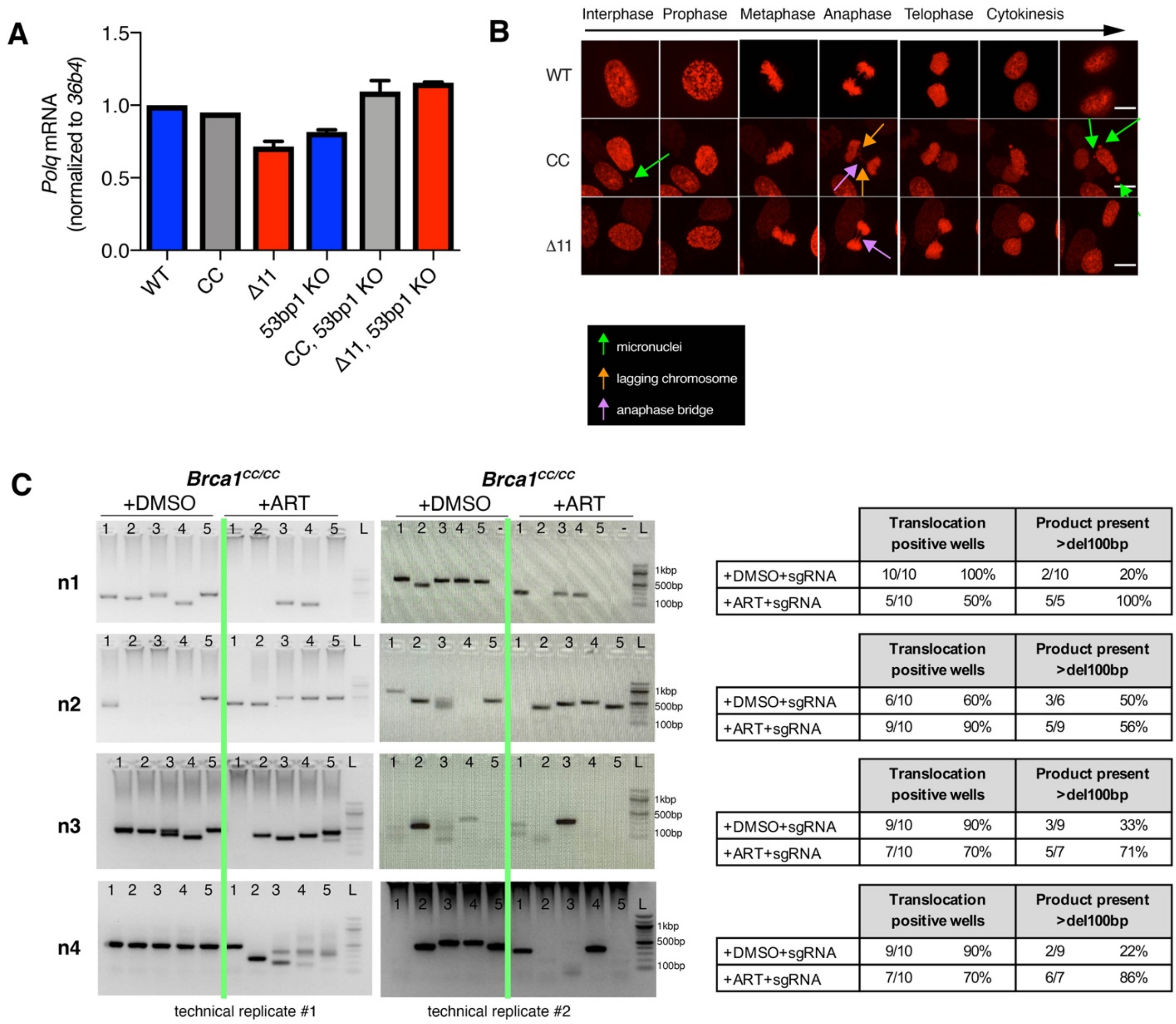

	Translocation positive wells		Product present >del100bp	
+DMSO+sgRNA	10/10	100%	2/10	20%
+ART+sgRNA	5/10	50%	5/5	100%

	Translocation positive wells		Product present >del100bp	
+DMSO+sgRNA	6/10	60%	3/6	50%
+ART+sgRNA	9/10	90%	5/9	56%

	Translocation positive wells		Product present >del100bp	
+DMSO+sgRNA	9/10	90%	3/9	33%
+ART+sgRNA	7/10	70%	5/7	71%

	Translocation positive wells		Product present >del100bp	
+DMSO+sgRNA	9/10	90%	2/9	22%
+ART+sgRNA	7/10	70%	6/7	86%

D

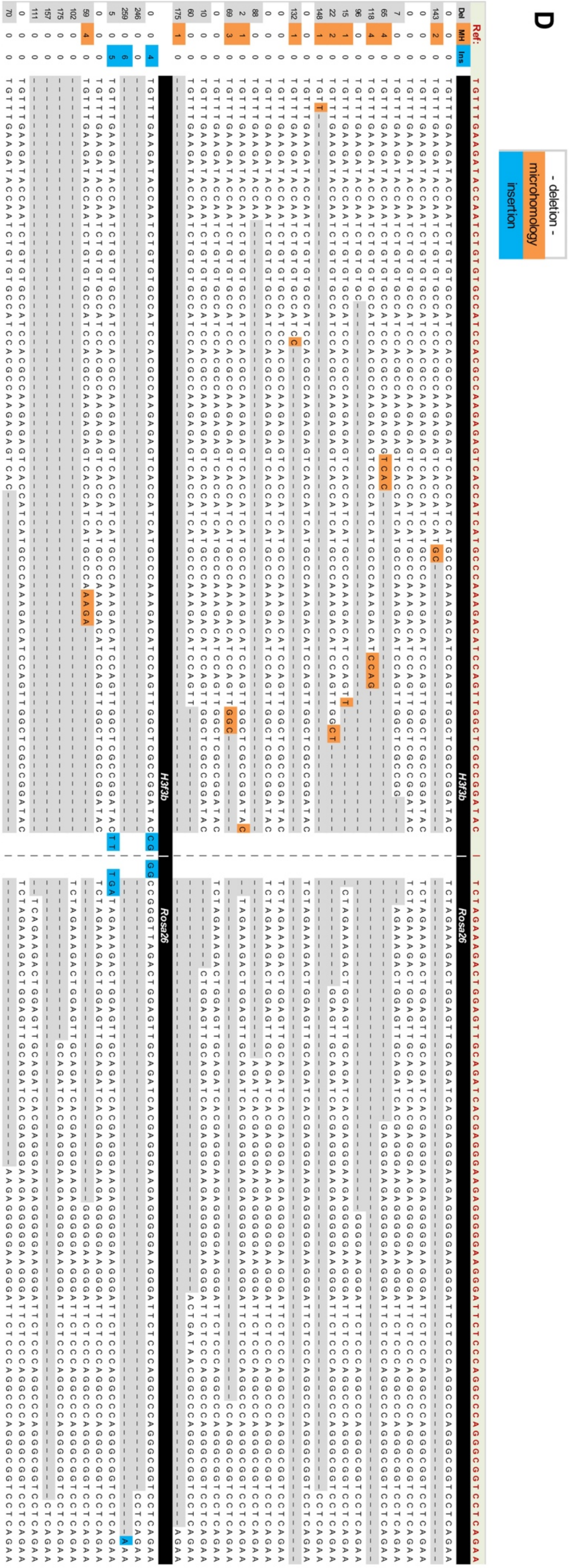
- deletion -
microhomology
insertion
Ref:
Del
MH
Ins
H3f3b
Rosa26

Supplementary Figure 3. Impact of Polθ activity in on mitosis and translocations

(**A**) RNA was extracted from MEFs with the indicated genotypes and assessed for *Polq* transcript levels by qRT-PCR and normalized to *36b4* expression. Mean and S.E.M. are shown.

(**B**) Representative images are shown from quantifications in **Fig. 5b**. $Brca1^{+/+}$, $Brca1^{CC/CC}$ and $Brca1^{\Delta 11/\Delta 11}$ MEFs expressing H2B-mCherry were observed using live cell imaging after 72h of DMSO or 10 μM ART558 treatments. Mitotic cells were tracked using maximum intensity projections and example of single cell progressions through mitosis in the presence of 10 μM ART558 are displayed. Examples of micronuclei (green arrows), lagging chromosomes (orange arrows), and anaphase bridges (pink arrows) are highlighted. Scale bar is equal to 10 μm.

(**C**) Agarose gels are shown for translocation assay quantifications in **Fig. 5d**. $Brca1^{CC/CC}$ expressing Cas9 were transduced with lentivirus encoding sgRNA to target Cas9 to the *Rosa26* and *H3f3b* loci. Cells were allowed 96h to repair breaks in the presence of 10 μM ART558 or DMSO and DNA extracted from cells in each well. 500 ng DNA from each well were used for nested PCR reactions with one primer adjacent to the *Rosa26* cut site and the second adjacent to the *H3f3b* cut site to amplify translocation repair products where the two loci have been fused. The nested PCR reactions for each well were run on agarose gels, where the presence of a 500bp band indicates the presence of the predicted translocation product. Of note, products less than the predicted band size were frequently detected, confirmed to a *Rosa26-H3f3b* translocation by sequencing, and quantified. Technical replicates were conducted and shown for each experiment. Each replicate was performed with independent 500ng DNA inputs from each well and yielded varied nested PCR results, indicating that unique translocations were present within each well at low frequency. In addition, 4 independent biological replicates were performed and gels shown. Table show interpretation of gels, >100 bp deletions are estimates based off band migration pattern. Bands were sequenced and those that produced single trace files that could be interpreted are reported in **Fig. 5D**.

(**D**) Nested PCR products from C were sequenced by Sanger sequencing, and deconvolved and aligned to the predicted junction (grey) using DECODR v3.0 software (decodr.org). Cropped regions of the aligned sequences are shown with microhomologies (orange), deletions (-), and insertions (blue) at the junctions highlighted. Source data are provided as a Source Data file.

Supplementary Figure 4.

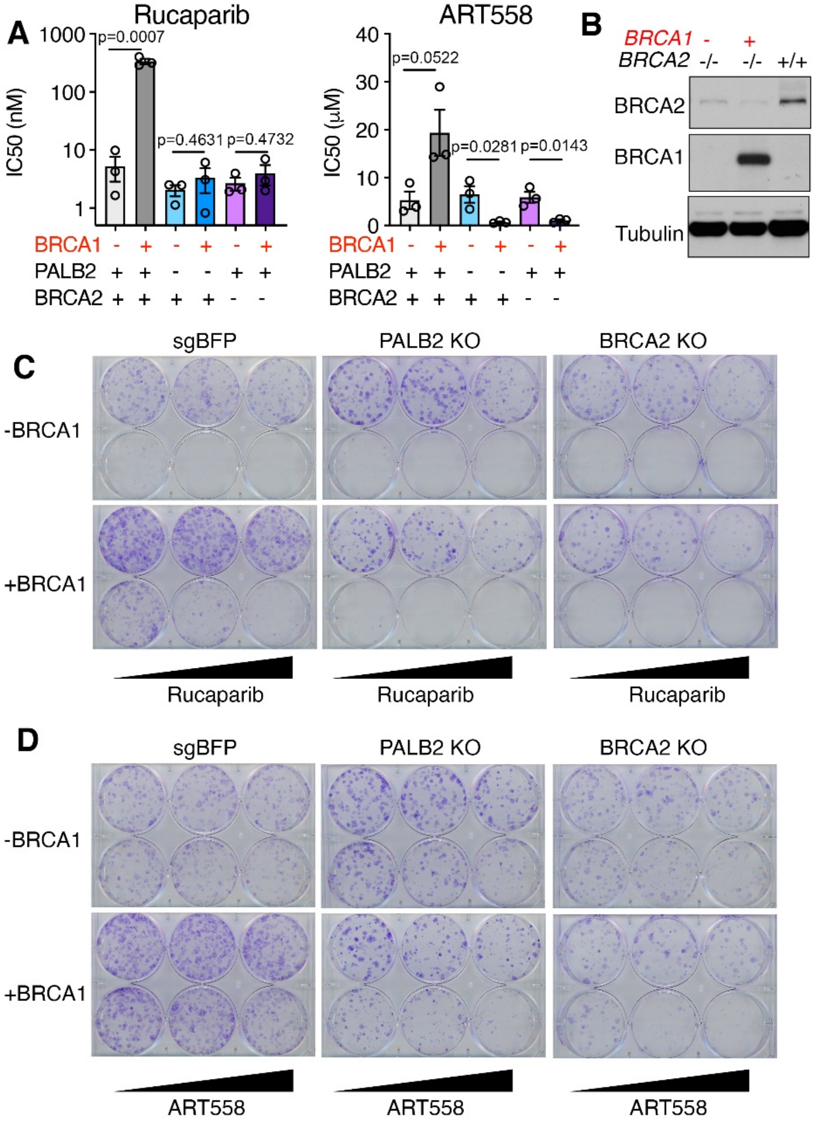

Supplementary Figure 4. Responses to PARPi and POLθ with distinct HR deficiencies

(**A**) BRCA1 null TNBC MDA-MB-436 cells with CRISPR/Cas9 mediated knockout of *PALB2*, *BRCA2* or with a BFP targeted control sgRNA, +/- BRCA1-Δ800, were seeded into increasing concentrations of rucaparib or ART558 and colony formation measured, shown in **Fig 6d,e**. IC50 values are shown as individual data points and mean and S.E.M. for n=3 independent experiments. Statistical significance was assessed for the indicated comparisons by unpaired, two-tailed *t*-tests and p values displayed.

(**B**) Western blot for the indicated proteins in MDA-MB-436 cells with CRISPR/Cas9 knockout of *BRCA2.*

(**C**) Representative plates are shown for rucaparib treatments 0nM, 1nM, 10nM, 100nM, 1000nM, 10000nM.

(**D**) Representative plates are shown for ART558 treatments 0μM, 0.1μM, 0.3μM, 1μM, 3μM, 10μM. Source data are provided as a Source Data file.

Supplementary Tables

Supplementary Table 1. Comparison of G-banding findings in MEF panel with *Polq*$^{+/+}$ and *Polq*$^{-/-}$

Genotype		Ploidy	Composite Karyotype[1]
Brca1$^{+/+}$	*Polq*$^{+/+}$	hexaploid to hyperhexaploid	108-135(6n),XXXXXX,-4,del(5)(E3),-6,-6,-7,-7,-9,-9,-10,-10,-11,-12,-13,+15,+15,-18, +3-17mar[cp6]
Brca1$^{+/+}$	*Polq*$^{-/-}$	hyperhexaploid	133-143(6n+),XXXXXX,+2,+2,+del(3)(F1)x2,+4,+4,+5,-6,del(6)(D)x2,+8,+9,+9, del(11)(B1),-12,-12,-12,+14,+14,+15,+15,-17,+6-10 mar
Brca1$^{CC/CC}$	*Polq*$^{+/+}$	triploid to tetraploid	64-75(4n), XXYY,-1,add(3)(H1),-4,add(5)(B),-6,-6,-7,-9,-10,-11,-12,-13,-14,-14, add(15)(A2)x2,-16,-17,-18,+5-13mar[cp7]
Brca1$^{CC/CC}$	*Polq*$^{-/-}$	near triploid	49-62(3n),add(X)(E),-Y or X,-1,-1,-2,add(2)(A2),-3,add(3)(A2),add(4)(A2),-5,-6,-7,-8,-9,-10,-11,-12,-13,-13,-14,-15,-16,-17, -17,-18,+19-27mar[cp8]
Brca1$^{\Delta11/\Delta11}$	*Polq*$^{+/+}$	near-triploid	56-65(3n),XXY,+1,+3,+3,ins(5)(B)x2,+8,+9,+10,-11,add(11)(E2),+12,+14,+15,+15,+16,+18,+19, +19,+6-9 mar
Brca1$^{\Delta11/\Delta11}$	*Polq*$^{-/-}$	hyperdiploid	42-49,XX,del(1)(H3),-3,-4,del(6)(F1),-8,-14,+15,-16,-18,+19,+9-14 mar

[1]Composite karyotypes are used when there is some variation from cell to cell in the abnormalities present. Only clonal abnormalities are reported in the composite karyotype.
