# Supplementary material for: Genetic separation of Brca1 functions reveal mutation-dependent Polθ vulnerabilities
Source: Nat Commun. 2023 Nov 24;14:7714. doi: 10.1038/s41467-023-43446-1 (PMC10673838; doi:10.1038/s41467-023-43446-1)
Supplement: Supplementary file 3 — Description of Additional Supplementary Files [file 41467_2023_43446_MOESM3_ESM.pdf]

Description of Additional Supplementary Files

File Name: Supplementary Data 1
Description: Insertion and deletion analysis of Sanger sequencing of clones following Cas9 targeting of *Polq*

File Name: Supplementary Data 2
Description: Rearrangement junctions in *Brca1*$^{CC/CC}$, *Polq*$^{+/+}$ (sgGFP) cells

File Name: Supplementary Data 3
Description: Rearrangement junctions in *Brca1*$^{CC/CC}$, *Polq*$^{-/-}$ cells
