# Supplementary material for: Genetic separation of Brca1 functions reveal mutation-dependent Polθ vulnerabilities
Source: Nat Commun. 2023 Nov 24;14:7714. doi: 10.1038/s41467-023-43446-1 (PMC10673838; doi:10.1038/s41467-023-43446-1)
Supplement: Supplementary file 7 — Reporting Summary [file 41467_2023_43446_MOESM7_ESM.pdf]

nature portfolio

Corresponding author(s): Neil Johnson, John Krais

Last updated by author(s): Oct 30, 2023

Reporting Summary

Nature Portfolio wishes to improve the reproducibility of the work that we publish. This form provides structure for consistency and transparency in reporting. For further information on Nature Portfolio policies, see our Editorial Policies and the Editorial Policy Checklist.

Statistics

For all statistical analyses, confirm that the following items are present in the figure legend, table legend, main text, or Methods section.

n/a	Confirmed	
☐	☒	The exact sample size (*n*) for each experimental group/condition, given as a discrete number and unit of measurement
☐	☒	A statement on whether measurements were taken from distinct samples or whether the same sample was measured repeatedly
☐	☒	The statistical test(s) used AND whether they are one- or two-sided<br>*Only common tests should be described solely by name; describe more complex techniques in the Methods section.*
☒	☐	A description of all covariates tested
☐	☒	A description of any assumptions or corrections, such as tests of normality and adjustment for multiple comparisons
☐	☒	A full description of the statistical parameters including central tendency (e.g. means) or other basic estimates (e.g. regression coefficient) AND variation (e.g. standard deviation) or associated estimates of uncertainty (e.g. confidence intervals)
☐	☒	For null hypothesis testing, the test statistic (e.g. *F*, *t*, *r*) with confidence intervals, effect sizes, degrees of freedom and *P* value noted<br>*Give P values as exact values whenever suitable.*
☒	☐	For Bayesian analysis, information on the choice of priors and Markov chain Monte Carlo settings
☒	☐	For hierarchical and complex designs, identification of the appropriate level for tests and full reporting of outcomes
☐	☒	Estimates of effect sizes (e.g. Cohen's *d*, Pearson's *r*), indicating how they were calculated

Our web collection on statistics for biologists contains articles on many of the points above.

Software and code

Policy information about availability of computer code

Data collection	Image collection and projection images were obtained from a Leica Stellaris 5 confocal microscope using Leica Application Suite X (LASX) software. mFISH data was collected using a Zeiss AxioImager Z2 fluorescence microscope with MetaSystems Isis/mFISH image analysis system.
Data analysis	Image analysis was performed using ImageJ (FIJI) version 2.1.0/1.53c. END-seq data was processed with Trimmomatic-0.36, BWa-0.7.17-r1188, Samtools-1.13, Picard-2.27.5, and Bedtools-v2.27.1. Statistical analysis and heatmap generation was performed in RStudio 1.3.1093 or with GraphPad Prism 9.

For manuscripts utilizing custom algorithms or software that are central to the research but not yet described in published literature, software must be made available to editors and reviewers. We strongly encourage code deposition in a community repository (e.g. GitHub). See the Nature Portfolio guidelines for submitting code & software for further information.

Data

Policy information about availability of data

All manuscripts must include a data availability statement. This statement should provide the following information, where applicable:

- Accession codes, unique identifiers, or web links for publicly available datasets
- A description of any restrictions on data availability
- For clinical datasets or third party data, please ensure that the statement adheres to our policy

All relevant data are available from the authors upon reasonable request. The data generated in this study are provided in the Source Data file. END-seq data are available at the GEO repository under accession number GSE244865. Mouse reference genome mm10 was downloaded from UCSC.

Research involving human participants, their data, or biological material

Policy information about studies with human participants or human data. See also policy information about sex, gender (identity/presentation), and sexual orientation and race, ethnicity and racism.

Reporting on sex and gender	N/A
Reporting on race, ethnicity, or other socially relevant groupings	N/A
Population characteristics	N/A
Recruitment	N/A
Ethics oversight	N/A

Note that full information on the approval of the study protocol must also be provided in the manuscript.

Field-specific reporting

Please select the one below that is the best fit for your research. If you are not sure, read the appropriate sections before making your selection.

☒ Life sciences ☐ Behavioural & social sciences ☐ Ecological, evolutionary & environmental sciences

For a reference copy of the document with all sections, see nature.com/documents/nr-reporting-summary-flat.pdf

Life sciences study design

All studies must disclose on these points even when the disclosure is negative.

Sample size	No statistical methods were used to pre-determine sample size. Most experiments were conducted with 3 independent replicates or as indicated in figure legends, selected based on standard practices.
Data exclusions	No data were excluded from analysis or reporting.
Replication	All experiments were reliably reproduced in independent experiments as stated in figure legends.
Randomization	Not applicable. No treatment assignments were performed.
Blinding	Not applicable. No treatment assignments were performed. Non-biased image analysis was performed using ImageJ.

Reporting for specific materials, systems and methods

We require information from authors about some types of materials, experimental systems and methods used in many studies. Here, indicate whether each material, system or method listed is relevant to your study. If you are not sure if a list item applies to your research, read the appropriate section before selecting a response.

Materials & experimental systems

n/a	Involved in the study
☐	☒ Antibodies
☐	☒ Eukaryotic cell lines
☒	☐ Palaeontology and archaeology
☐	☒ Animals and other organisms
☒	☐ Clinical data
☒	☐ Dual use research of concern
☒	☐ Plants

Methods

n/a	Involved in the study
☒	☐ ChIP-seq
☒	☐ Flow cytometry
☒	☐ MRI-based neuroimaging

Antibodies

Antibodies used

Immunofluorescence:
gH2ax (Millipore Sigma, 05-636 lot 3959058, 1:1000)
Rad51 (Abcam, ab133534 lot GR219215-47, 1:50000)
Rpa32 (Cell Signaling, 2208 lot 5, 1:1000 for MEFs or Millipore Sigma, NA18 lot 3296156, 1:1000 for human cells)

Western blot (mouse samples):
Brca1 (R&D Systems, MAB22101 lot CBYA0121031, 1:1000)
53bp1 (Novus Biologicals, NB100-305SS lot G, 1:1000)
gH2ax (Millipore Sigma, 05-636 lot 3959058, 1:1000)
Tubulin (Cell Signaling, 2148 lot 8, 1:2000)

Western blot (human samples):
BRCA1(Millipore Sigma, OP92 lot 3911114, 1:500)
PALB2 (Bethyl Laboratories, A301-246A lot 2, 1:2000)
53BP1 (Millipore Sigma, MAB3802 lot 3834817, 1:1000)
Tubulin (Cell Signaling, 2148 lot 8, 1:2000)

Validation

Appropriate positive and negative controls were included in the experimental design to confirm the antibodies were specific. Additional validation information is below:

gH2ax (Millipore Sigma, 05-636) - validation stated at https://www.emdmillipore.com/US/en/product/Anti-phospho-Histone-H2A.X-Ser139-Antibody-clone-JBW301,MM_NF-05-636

Rad51 (Abcam, ab133534) - validation stated at https://www.abcam.com/products/primary-antibodies/rad51-antibody-epr40303-ab133534.html

Rpa32 (Cell Signaling, 2208) - validation stated at https://www.cellsignal.com/products/primary-antibodies/rpa32-rpa2-4e4-rat-mab/2208

Rpa32(Millipore Sigma, NA18) - validation stated at https://www.emdmillipore.com/US/en/product/Anti-Replication-Protein-A-Ab-2-Mouse-mAb-RPA34-19,EMD_BIO-NA18

Brca1 (R&D Systems, MAB22101) - validation stated at https://www.rndsystems.com/products/human-mouse-brca1-c-terminus-antibody-440621_mab22101

53bp1 (Novus Biologicals, NB100-305SS) - validation stated at https://www.novusbio.com/products/53bp1-antibody_nb100-305

BRCA1(Millipore Sigma, OP92) - validation stated at https://www.emdmillipore.com/US/en/product/Anti-BRCA1-Ab-1-Mouse-mAb-MS110,EMD_BIO-OP92

PALB2 (Bethyl Laboratories, A301-246A) - validation stated at https://www.thermofisher.com/antibody/product/PALB2-Antibody-Polyclonal/A301-246A

53BP1 (Millipore Sigma, MAB3802) - validation stated at https://www.emdmillipore.com/US/en/product/Anti-53BP1-Antibody-clone-BP13,MM_NF-MAB3802

Tubulin (Cell Signaling, 2148) - validation stated at https://www.cellsignal.com/products/primary-antibodies/a-b-tubulin-antibody/2148

Eukaryotic cell lines

Policy information about cell lines and Sex and Gender in Research

Cell line source(s)	MDA-MB-436 cell lines were obtained from ATCC. HEK293T were purchased from Takara Bio. Mouse embryonic fibroblast cell lines were generated.
Authentication	MDA-MB-436 cells were confirmed by short tandem repeat (STR) profiling using IDEXX analysis. Genotyping was used to confirm identities of MEF cell lines as described in the methods section. HEK293T cells were used within 10 passages from the manufacturer and not authenticated.
Mycoplasma contamination	Cell lines were confirmed negative for mycoplasma contamination by Lonza MycoAlert assay LT07-705.
Commonly misidentified lines (See ICLAC register)	No commonly misidentified lines were used in this study.

Animals and other research organisms

Policy information about studies involving animals; ARRIVE guidelines recommended for reporting animal research, and Sex and Gender in Research

Laboratory animals	Polq and 53bp1 mutant mice were obtained from the Jackson Laboratory. Brca1-d11 mice were obtained from NCI. Brca1-CC mice were previously generated in our lab (Nacson et al, Mol Cell 2020). Mouse crosses were performed on a mixed background using males and females less than 6 months old and were housed in standard conditions approved by the American Association for Accreditation of Laboratory Animal Care and the Fox Chase Cancer Center Institutional Animal Care and Use Committee.
Wild animals	No wild animals were used in this study.
Reporting on sex	Sex-based analysis was not performed in this study.
Field-collected samples	No field-collected samples were used in this study.
Ethics oversight	The Fox Chase Cancer Center (FCCC) Institutional Animal Care and Use Committee (IACUC) approved experiments involving mice.

Note that full information on the approval of the study protocol must also be provided in the manuscript.
